# Genomic Regions Associated with Milk Composition and Fertility Traits in Spring-Calved Dairy Cows in New Zealand

**DOI:** 10.3390/genes14040860

**Published:** 2023-04-01

**Authors:** J. M. D. R. Jayawardana, Nicolas Lopez-Villalobos, Lorna R. McNaughton, Rebecca E. Hickson

**Affiliations:** 1 School of Agriculture and Environment, Massey University, Palmerston North 4410, New Zealand; d.jayawardana@massey.ac.nz; 2 Department of Animal Science, Faculty of Animal Science and Export Agriculture, Uva Wellassa University, Badulla 90000, Sri Lanka; 3 Livestock Improvement Corporation, Private Bag 3016, Hamilton 3240, New Zealand; 4 Focus Genetics, 17C Mahia St, Ahuriri, Napier 4144, New Zealand

**Keywords:** genome-wide association study, milk composition, fertility, dairy cattle, candidate gene

## Abstract

The objective of this study was to identify genomic regions and genes that are associated with the milk composition and fertility traits of spring-calved dairy cows in New Zealand. Phenotypic data from the 2014–2015 and 2021–2022 calving seasons in two Massey University dairy herds were used. We identified 73 SNPs that were significantly associated with 58 potential candidate genes for milk composition and fertility traits. Four SNPs on chromosome 14 were highly significant for both fat and protein percentages, and the associated genes were *DGAT1, SLC52A2, CPSF1,* and *MROH1*. For fertility traits, significant associations were detected for intervals from the start of mating to first service, the start of mating to conception, first service to conception, calving to first service, and 6-wk submission, 6-wk in-calf, conception to first service in the first 3 weeks of the breeding season, and not in calf and 6-wk calving rates. Gene Ontology revealed 10 candidate genes (*KCNH5, HS6ST3, GLS, ENSBTAG00000051479, STAT1, STAT4, GPD2, SH3PXD2A, EVA1C*, and *ARMH3*) that were significantly associated with fertility traits. The biological functions of these genes are related to reducing the metabolic stress of cows and increasing insulin secretion during the mating period, early embryonic development, foetal growth, and maternal lipid metabolism during the pregnancy period.

## 1. Introduction

New Zealand dairy farming is pasture-based with cows calving in winter (June/July) in each production season. To retain a seasonal calving pattern, dairy farmers aim to maximise the proportion of cows mated and in-calf early in the breeding season to generate more days in milk. In New Zealand, the most common breeds used in dairy farming are Holstein Friesian (F; 32.5%), Jersey (J; 8.2%), and a crossbred of Holstein Friesian and Jersey (F × J; 49.6%) [1]. The milk production of New Zealand dairy cows has increased in recent production seasons due to improvements in management practices and genetic selection for milk production [1]. Since 2001, cow fertility has incorporated national genetic evaluation in New Zealand [2,3]. The New Zealand national averages for 3-wk submission (SR21), conception to first service (PRFS), 6-wk in-calf (PR42) were 81.3%, 52.7%, and 67.7% in the 2020–2021 production season, respectively [1]. Kerslake et al. [4] reported that reproductive failure is the primary cause of cow wastage in New Zealand dairy herds. About 31% of the annual cost of wastage (NZD 7279/100 cows) is associated with cow removal due to the failure to conceive or maintain a pregnancy.

Heritability estimates for fertility traits are low in New Zealand dairy cows (<10%) and antagonistic genetic correlations have been reported between production and fertility traits in New Zealand dairy cows [3,5,6]. The simultaneous improvement of milk production and fertility is difficult to achieve in dairy cattle breeding due to this antagonistic relationship. In this context, genomic selection can be used in dairy cattle breeding programs to improve the rate of genetic gain by increasing the accuracy of genomic breeding values [7,8,9] and reducing the generation intervals [10]. Genomic selection uses the information of genome-wide single nucleotide polymorphisms (SNPs) markers. Ma, et al. [11] reported that the development of genomic selection on fertility traits has stabilised and even reversed the declining trend of cow fertility. 

Genome-wide association studies (GWAS) are a powerful tool for detecting genomic regions that explain the genetic variation of phenotypic traits. Identification of fertility-associated quantitative trait loci (QTL) would support the efficiency of genomic selection for fertility traits. Previous studies have identified several QTL regions and functional candidate genes associated with the milk composition and fertility traits of dairy cows in several countries [12,13,14,15,16]. Since 2008, genomically enhanced breeding values have been used for genomic selection in New Zealand dairy cattle [17,18].

In New Zealand, cows that produce milk with more fat and protein are more profitable for dairy farmers because the payment system rewards yields of fat and protein and penalises milk volume. It is important to identify the genomic regions and genes that are associated with fat and protein percentages to improve the genomic breeding values in those traits. The most important measures of the fertility performance of dairy cows are SR21, PR42, and the not in calf rate (NIC) at the end of the breeding season. However, no published GWAS were found in the literature reporting candidate genes associated with SR21, PR42, and NIC. There is little information available on GWAS for fertility traits in spring-calved dairy cows in New Zealand. Though the fertility performance of New Zealand dairy cattle is higher than the Irish, Australian, United Kingdom, and USA dairy cattle populations [19], it is well below New Zealand industry targets (SR21 = 90%, PRFS = 60% and PR42 = 78%) [20], and potentially, the existing fertility breeding values could be enhanced with the use of genomic information.

The objective of the present study was to identify the SNPs and candidate genes affecting milk composition and fertility traits in spring-calved dairy cows milked in Massey University dairy herds in New Zealand.

## 2. Materials and Methods

### 2.1. Data

Data from Massey University Dairy 1 and Dairy 4 herds, Palmerston North, New Zealand, were used for this study. Calving, mating, pregnancy diagnosis, herd testing milking dates, lactation yields, and pedigree records of spring-calved dairy cows were extracted from the Livestock Improvement Corporation (LIC), New Zealand. The initial dataset consists of 6931 records of 2270 cows from the 2014–2015 and 2021–2022 calving seasons. Both herds had at least three herd testing milk records per lactation during the study period. The breed distribution was 129 F, 117 J, and 239 F × J cows in Dairy 1 and 430 F, 23 J, and 696 F × J cows in Dairy 4. The management of the cows in Dairy 1 includes full-lactation once-daily milking with minimal supplementary feeding and a low stocking density (2.1 cows/ha). In contrast, the Dairy 4 herd is milked twice daily, with higher levels of supplementary feeding and a greater stocking density (2.8 cows/ha). Data from calving season 2014–2015 in the Dairy 1 herd were excluded from the present analysis because it was the transitional year of changing from milking twice daily to once daily.

### 2.2. Phenotypic Traits

The milk composition and fertility traits investigated in this study are presented in Table 1. 

Fat percentage (FP), protein percentage (PP), and lactose percentage (LP) were calculated for each lactation record as the ratio between fat yield, protein yield, or lactose yield and milk yield multiplied by 100. 

The mating start date and mating end date for each calving season were sourced from the LIC database. Cows (*n* = 161) with mating records outside the mating season in the Dairy 4 herd in the calving season of 2018–2019 were excluded for fertility traits calculation. Those cows were used for other experiments that demanded another mating strategy.

The average mating length of the breeding season was 71.0 days in Dairy 1 and 69.5 days for Dairy 4. Cows (*n* = 328) that were missing both a pregnancy diagnosis (PD) result and all herd tests after the end of the breeding period were assumed to have left the herd prior to the end of the mating period and were excluded from the analysis. Pregnancy diagnosis results where the foetal age was estimated and where cows were tested between 35–122 days (inclusive) from conception were used. Conception dates were calculated as the date of PD minus the estimated foetal age for cows with a pregnancy status of ‘pregnant’. In some cases, cows (*n* = 63) with positive PD had estimated pregnancy day counts outside the bounds (<35 days or >122 days) or no estimated foetal age results but calved in the subsequent season. In such cases, conception dates were calculated as subsequent calving dates minus the gestation length of 282 days. The conception date was not calculated for cows (*n* = 16) with a pregnancy status of ‘pregnant’ but without either estimated foetal age or subsequent calving date. These cows were culled due to mastitis, low production, and udder problems. 

Cows (*n* = 29) with no recorded artificial breeding (AB) inseminations were retained with a penalty date in the analysis. The penalty date was assigned for the first service date as the end of the AB period in each herd within the calving season. The end of the AB period was defined for each herd as the date of the last AB insemination that was not followed by another AB insemination within 7 days [21]. Likewise, cows that had not conceived (*n* = 787) at the end of the breeding season were included, with a penalty date for conception as the mating end date in each herd plus 21 days [5,22]. The calving interval (CI) was calculated for cows that did not calve in the subsequent calving season but had a positive pregnancy diagnosis (*n* = 1113). The calving date was assigned as the conception date plus the gestation length of 282 days and the CI was calculated as the assigned calving date minus the calving date in the respective season.

Submission in the first 21 days (SR21) or 42 days (SR42) of the mating season was coded as 1 if a cow had at least one AB record in the first 21 days or 42 days from the start of the mating date, respectively, and 0 otherwise. Pregnant by 21 days (PR21) or 42 days (PR42) was coded as 1 if a cow was pregnant in the first 21 days or 42 days of from the mating start date, respectively, otherwise 0. Only cows whose first service was to AB and within first 21 days from the mating start date were used to determine pregnant to first service (PRFS), which was classified as 1 for cows whose date of first service coincided with their date of conception, and 0 otherwise. Not in calf (NIC) at end of the breeding season was coded as 1 for cows with the last PD outcome ‘empty’ and 0 for cows with the last PD outcome ‘pregnant’. Cows (*n* = 18) with the last pregnancy status ‘doubtful’ without subsequent calving were also coded as 1. The planned start of calving (PSC) date was calculated for a herd by adding 282 days to the herd’s mating start date in each calving season. If a cow calved less than 21 days or 42 days after the PSC, then 3-wk calving (CR21) or 6-wk calving (CR42) was coded as 1, otherwise 0.

The final phenotypic dataset comprised 6382 records. Five parity classes were defined, cows in their first four parities were considered separately and a parity number of five or higher was grouped into one class. The heterosis coefficient for each cow was calculated using the following equation [23]:

(1) $$h_{F\times J}=\alpha_{F}^{S}\alpha_{J}^{D}+\alpha_{J}^{S}\alpha_{F}^{D}$$

where $h_{F\times J}$ is the coefficient of heterosis between F and J in the progeny; $\alpha_{F}^{S}$ and $\alpha_{J}^{S}$ are the breed proportions of F and J in the sire; and $\alpha_{J}^{D}$ and $\alpha_{F}^{D}$ are the breed proportions of J and F in the dam, respectively.

### 2.3. Descriptive Statistics

Descriptive statistics for milk production and fertility traits were obtained using the MEANS procedure of SAS package 9.4 (SAS Institute Inc. 2013, Cary, NC, USA).

### 2.4. Genotypes and Quality Control

DNA was extracted from ear punch tissue samples for genotyping with Illumina Bovine Illumina 50K SNP-chips. The initial genotype data consisted of 1774 cows with 132,154 SNPs during the study period. The SNP & Variation Suite (SVS 8.8 [24]) software was used for quality control. The genotypes recorded in Illumina A/B allele format were converted to 0, 1, or 2, depending on the number of B alleles present at each locus. In the filtering process firstly, SNPs with a call rate ≤ 80% were excluded. Then, individuals with a call rate ≤ 80% or with a minor allele frequency (MAF) ≤ 5% and significant deviation from Hardy–Weinberg Equilibrium (HWE) *p* values (*p* < 10^−6^) were excluded from the dataset. After the quality control steps, a total of 1537 individuals with 42,667 SNPs were selected for association analysis.

### 2.5. Genome-Wide Association Analyses

The phenotypes used for the GWAS were pre-corrected using fixed effects, covariates, and random effects using the ASReml 4.1 software package [25]. A single-trait animal repeatability model was used to adjust repeated measures in the phenotypes. The models included the fixed effects of herd-year as a contemporary group, parity (1, 2, 3, 4, ≥5), the regression coefficient associated with the proportion of F, the regression coefficient associated with heterosis, and the regression coefficient associated with deviation of the calving date from the median calving date of the herd within the season as a covariate and the random effect of the animal, cow the permanent environment effect, and the random residual.

A genome-wide association analysis (GWAS) using a mixed linear model was used to test associations between individual SNP traits using GCTA software [26]. 

The following model was fitted for each trait:

(2) y=μ+Xβ+g+e

where **y** was the pre-corrected phenotype for the cow, **μ** was the overall mean, **β** was the fixed effect of the candidate SNP to be tested for the association, **X** was the SNP genotype indicator variable coded as 0, 1, or 2, **g** was the random effect that captures the polygenic effect of the other SNPs with $g\sim N\left(0,G\sigma_{g}^{2}\right)$, where **G** was the genomic relationship matrix between the cows and $\sigma_{g}^{2}$ was the additive genetic variance explained by markers, and **e** was the random residual effect with $e\sim N\left(0,I\sigma_{e}^{2}\right)$, where **I** was the identity matrix of order n = 1537 and $\sigma_{e}^{2}$ was the residual variance. The variance of **y** was assumed as $var\left(y\right)=G\sigma_{g}^{2}+I\sigma_{e}^{2}$. Diagonal and off-diagonal values of **G** were calculated as follows:

(3) $$G_{jk}=\frac{1}{m}\sum_{i}G_{ijk}$$

(4) $$=\left\{\begin{array}{c} 1+\frac{1}{m}\sum_{i=1}^{m}\frac{x_{ij}^{2}-\left(1+2p_{i}\right)x_{ij}+2p_{i}^{2}}{2p_{i}(1-p_{i})},j=k\\ \frac{1}{m}\sum_{i=1}^{m}\frac{\left(x_{ij}-2p_{i})(x_{ik}-2p_{i}\right)}{2p_{i}(1-p_{i})},\;j\neq k \end{array}\right.$$

where $G_{ijk}$ was the estimated genomic relationship between animal j and k at locus i and m was the total number of SNPs (m = 42,667), $x_{ij}$ and $x_{ik}$ were genotypes with the number of copies for reference of the copies and for reference of the allele for the ith SNP jth and kth animal, and $p_{i}$ was the allele frequency of the allele for which the homozygous genotype was coded as 2.

The estimated associations were represented in Manhattan plots in which −log_10_ (*p*-values) were plotted against the genomic locations of the markers using qqman package in R software 4.2.1 [27]. The significance threshold values for the GWAS were estimated using Bonferroni multiple-test correction, which adjusts *p*-values due to the increased risk of a type I error when making multiple statistical tests [28]. The Bonferroni-corrected genome-wide significance threshold was estimated as 0.05/m (0.05/42,667 = 1.17 × 10^−6^), which was 5.93 on a −log_10_ (*p*-value) scale. Bonferroni correction was considered too conservative; therefore, the suggestive significance threshold was also estimated as 1/m. The *p*-value threshold for suggestive associations was 2.34 × 10^−5^ (1/42,667), which corresponded to 4.63 in the −log_10_(*p*-value) scale. The genomic position, allele substitution effect and their standard errors, and the closest gene were described for the SNPs that exceeded the Bonferroni-adjusted *p*-value thresholds.

### 2.6. Candidate Genes and Functional Analysis

Potential candidate genes were explored using Ensembl (release 107) (http://www.ensembl.org/index.html, accessed on 10 September 2022) based on the Bos taurus reference genome in ARS-UCD1.2 genome assembly [29]. The individual significant SNPs for each trait were examined to locate the closest genes within 100 kb upstream and downstream from the identified SNP. Gene annotation was recorded if the gene was positioned 5 kb upstream or downstream from the gene boundaries. The biological functions of the associated candidate genes were reviewed using the Gene Ontology (GO) tool in Ensembl.

## 3. Results

### 3.1. Descriptive Statistics

Descriptive statistics for the milk composition and fertility traits of the selected cow population are presented in Table 2.

The cows produced on average a 4968 L milk yield, 232 kg fat yield, 188 kg protein yield, and 251 kg lactose yield during the study period. The average intervals for the first AB and conception after the herd’s start of mating dates were 10.9 days and 29.6 days, respectively. A higher proportion of cows were mated within the first 3 weeks and 6 weeks of the mating season (91% and 99%, respectively). Fifty-four % and seventy-six % of the cows conceived in the first 3 weeks and 6 weeks of the mating seasons, respectively, and forty-eight % were pregnant to their first service. Not in calf at the end of the breeding seasons was 12%. The calving by the first 3 weeks and 6 weeks from the PSC was 68% and 91%, respectively.

### 3.2. GWAS for Milk Composition Traits

**Figure 1:**
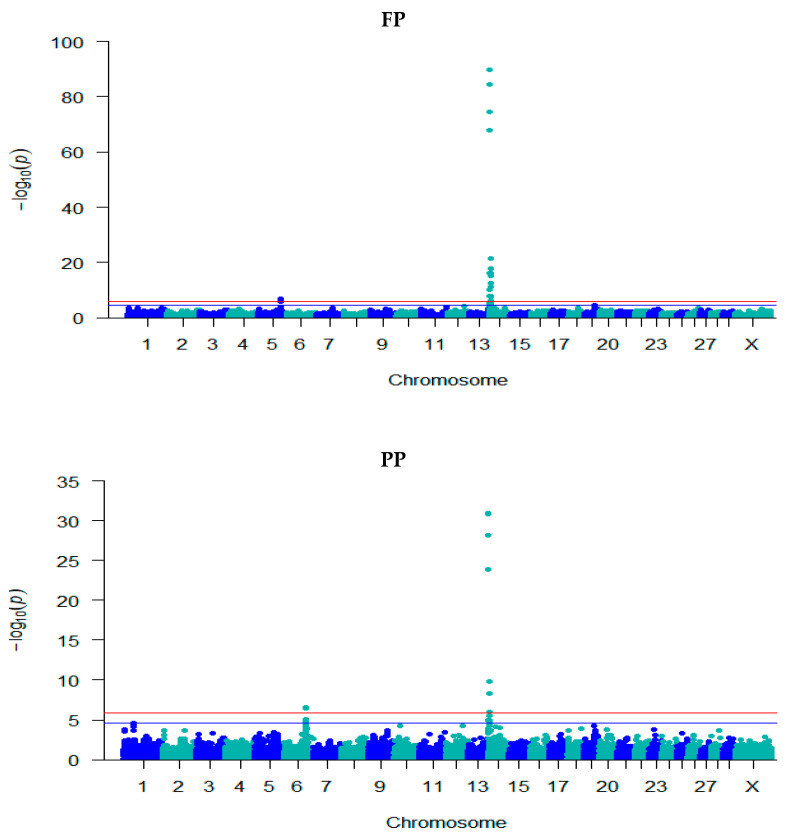

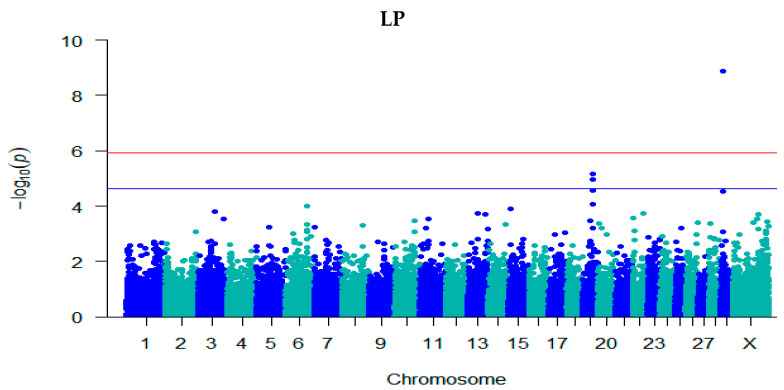
Manhattan plots for −log_10_ *p*-values of marker effects for the milk composition traits of fat percentage (**FP**), protein percentage (**PP**), and lactose percentage (**LP**). The genome-wide significance threshold of Bonferroni correction is represented by the red line at −log_10_ *p*-value = 5.93, and the suggestive significance threshold is represented by the blue line at −log_10_ *p*-value = 4.63.

Figure 1 shows the Manhattan plots for fat, protein, and lactose percentages. The SNPs that surpassed the genome-wide significance *p*-value threshold (*p* < 1.17 × 10^−6^) with position, allele substitution effect, gene annotation, and candidate gene name are presented in Table 3.

A total of 33 SNPs met the genome wide significance threshold, and they were across the traits FP, PP, and LP. According to the results, 19 SNPs from chromosomes 5 and 14 were significantly associated with FP, 13 SNPs from chromosomes 6 and 14 were significantly associated with PP, and one SNP on chromosome 29 was significantly associated with LP at genome-wide significance threshold. The majority of associations were found on chromosome 14 for both FP and PP. The four top SNPs on chromosome 14 (14:1,736,599, 14:1,765,835, 14:1,801,116, and 14:1,880,378) were highly significant for both FP and PP, with genome wide significance levels of −log10 (*p*) = 74.5, 84.4, 89.8, and 68.0 for FP and 28.1, 30.8, 31.0, and 23.9 for PP. 

The associated SNP markers were annotated to 26 potential candidate genes for FP, PP, and LP at a genome-wide significance level. For both FP and PP, the majority of significant SNPs were mapped to introns (39%), followed by the intergenic regions (21%), missense variants (15%), upstream regions closest to the candidate genes (12%), synonymous variants (9%) and 3 prime UTR (3%). The highly significant peak on chromosome 14 mapped to the *DGAT1* gene for FP and PP. The four most significant SNPs for both FP and PP that are located on chromosome 14 introduce a missense variant in the *CPSF1* gene, a synonymous variant in the *SLC52A2* gene, and an intron of the *DGAT1* and *MROH1* genes, respectively. Likewise, three significant SNPs on chromosome 14 are located within the intron of the *IQANK1, ZC3H3*, and *RHPN1* genes for both FP and PP. The SNPs that are positioned on 14: 1,653,693, 14: 1,757,935, 14: 2,826,632, and 14: 4,078,923 were annotated upstream of genes *FOXH1, ADCK5, LYPD2,* and *AGO2*, respectively. 

Two significant SNPs (5: 93,945,738 and 5: 93,949,810) on chromosome 5 for FP were found in the *MGST1* gene, located within an intron region of the *MGST1* gene. Likewise, four significantly associated SNPs (6:87,390,576, 6:8,7390,612, 6:87,390,673, and 6:87,390,681) on chromosome 6 for PP mapped to *CSN3* and those are located on the missense, synonymous, and 3 prime UTR of the *CSN3* gene. 

Although there were strong peaks on chromosome 14 for both FP and PP, none of the SNPs on chromosome 14 were associated with LP. The single SNP detected a genome widely significant for LP on chromosome 29 and located within the intron of the gene *PICALM*. In addition, one peak on chromosome 19 was observed and two SNPs on this region were suggestively significant. The SNPs that surpassed the suggestive significance *p*-value threshold (*p* < 2.34 × 10^−5^) are explained in the Supplementary Materials (Table S1).

### 3.3. GWAS for Fertility Traits

**Figure 2:**
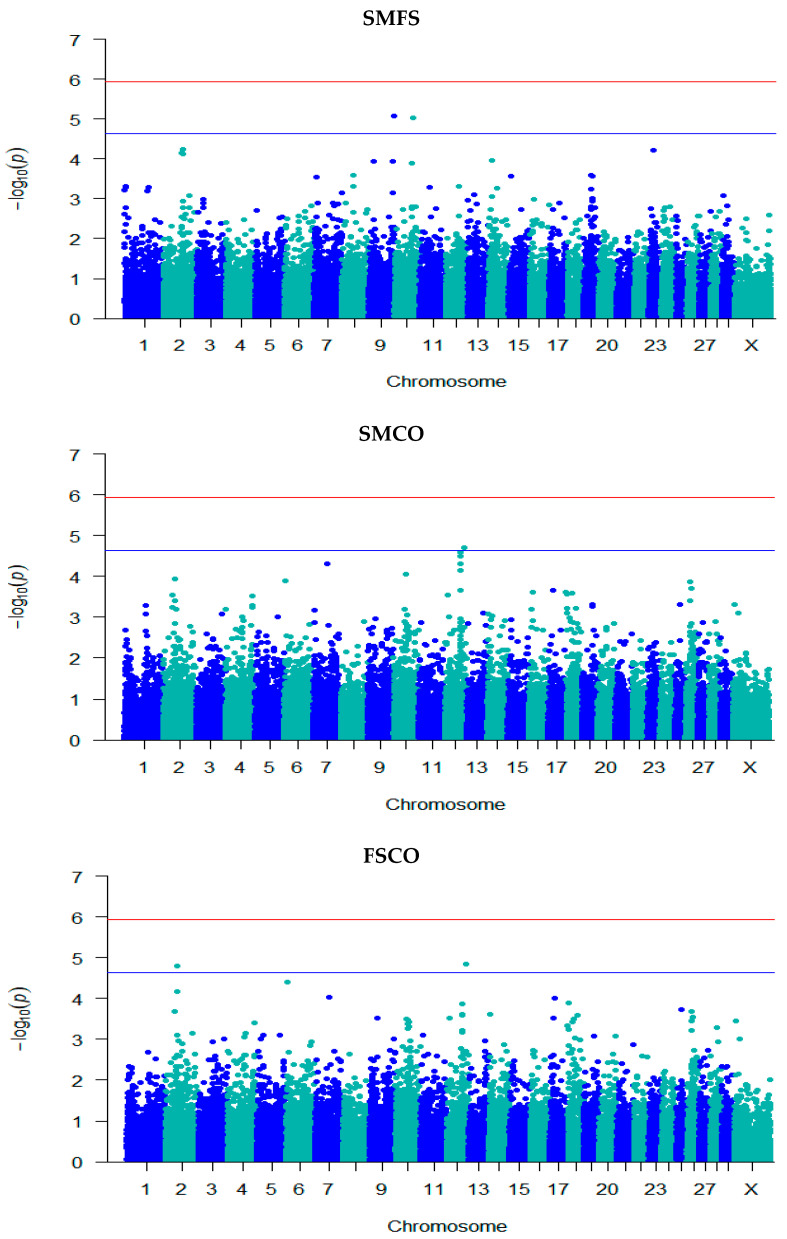

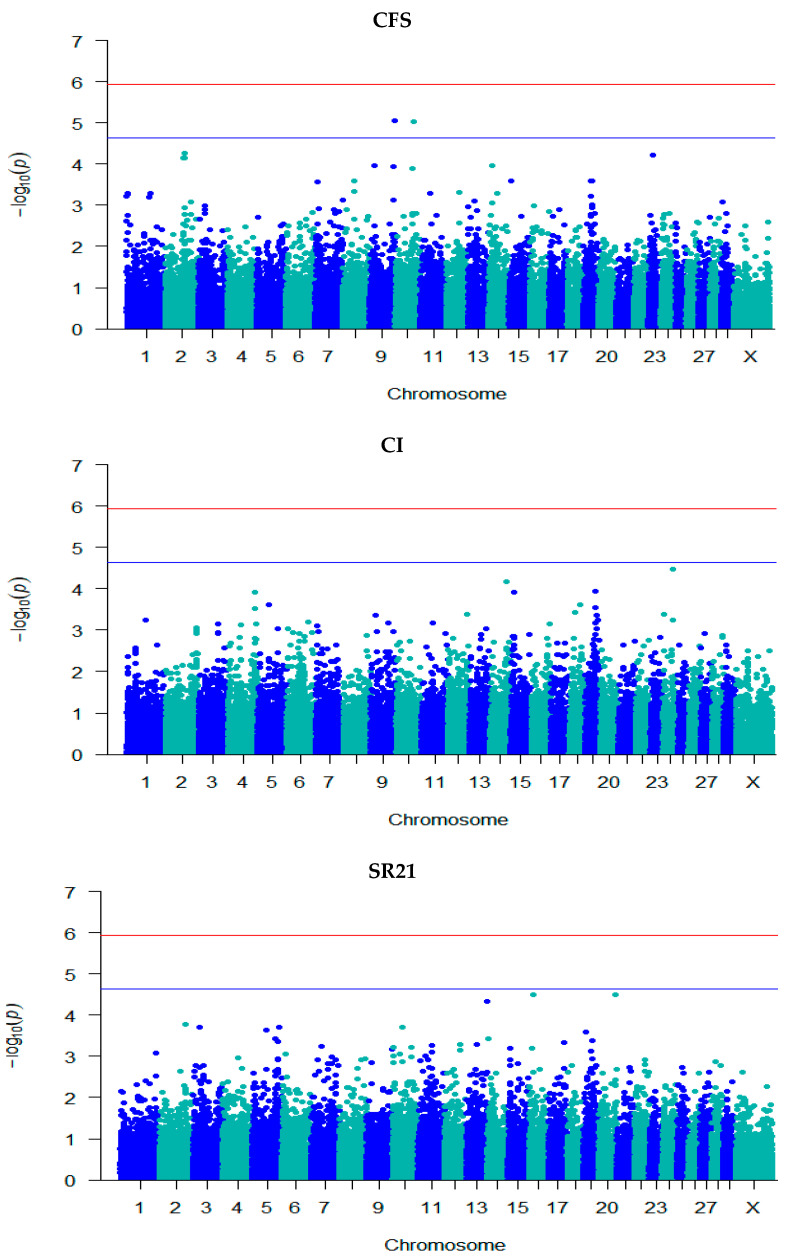

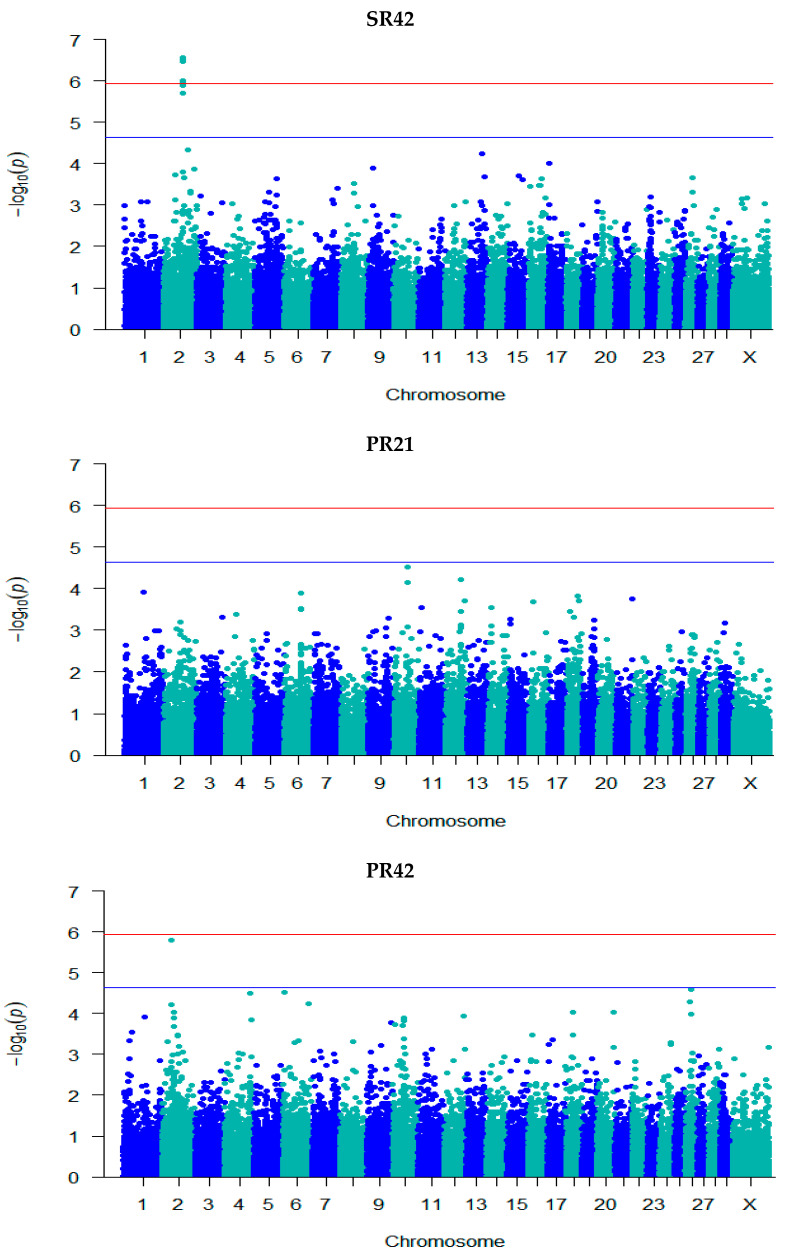

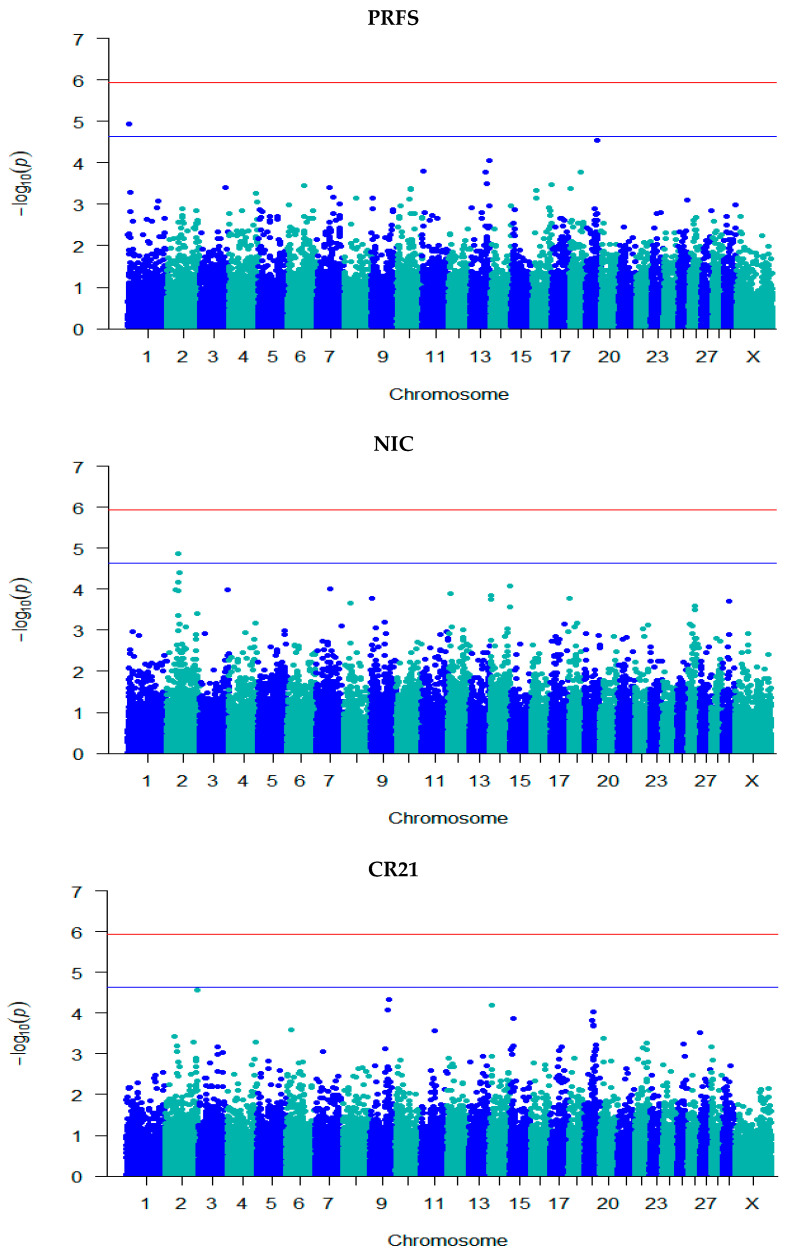

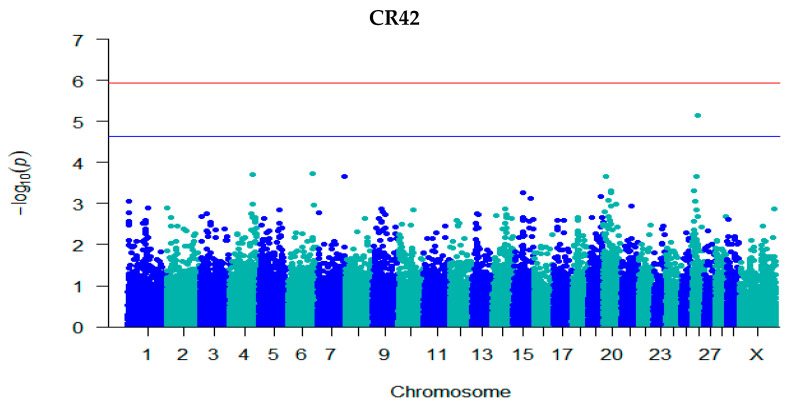
Manhattan plots for −log_10_ *p*-values of marker effects for the interval from the start of mating to first service (SMFS), the interval from the start of mating to conception (SMCO), the interval from first service to conception (FSCO), the interval from calving to first service (CFS), consecutive calving intervals (CI), 3 weeks submission (SR21), 6 weeks submission (SR42), 3 weeks in-calf (PR21), 6 weeks in-calf (PR42), pregnant to first service in the first 3 weeks of the breeding season (PRFS), not in calf at end of the breeding season (NIC), 3 weeks calving (CR21), and 6 weeks calving rate (CR42). The genome-wide significance threshold of Bonferroni correction is represented by the red line at −log_10_ *p*-value = 5.93, and the suggestive significance threshold is represented by the blue line at −log_10_ *p*-value = 4.63.

The Manhattan plots from the GWAS with fertility traits are shown in Figure 2 and the SNPs that surpassed both genome-wide and suggestive significance thresholds are presented in Table 4 with the position, allele substitution effect, gene annotation, and candidate gene name.

Genome-wide significant associations were found only with the SR42 phenotype. Seven genome-wide significant SNPs (2:79,474,217, 2:79,486,672, 2:79,706,385, 2:79,817,588, 2:79,908,334, 2:79,946,595, and 2:79,975,164) were detected on chromosome 2 for SR42, which are located in intron variants within the genes *GLS, ENSBTAG00000051479, STAT1,* and *STAT4*. In total 22 SNPs were found to be associated with SMFS, SMCO, FSCO, CFS, SR42, PR42, PRFS, NIC, and CR42 at the suggestive significant threshold and the most significant SNPs for fertility traits were detected on chromosome 2. The association analysis did not detect any significant SNPs for the traits CI, SR21, PR21, and CR21 either at genome-wide significance or suggestive significance levels. 

The significantly associated SNPs were annotated to 10 potential candidate genes on chromosomes 1, 2, 9, 10, 12, and 26. Most of the suggestive significant SNPs for fertility traits were mapped to introns (59%), 36% were mapped to intergenic regions and the remaining 5% were located upstream closest to the genes. The individual SNP 10:75,105,774 was significantly associated with SMFS and CFS and located within intron of the gene *KCNH5*. Likewise, the SNP at position 12:77,611,452 was significantly associated with both the SMCO and FSCO and mapped within the intron of the gene *HS6ST3*. Two SNPs on chromosome 2 (2: 39,681,141) and 26 (26: 24,432,758) were detected as significant for PR42, which are positioned within the intron of the gene *GPD2* and upstream of the gene *SH3PXD2A*, respectively. One significant SNP (1: 2,262,097) was identified for PRFS and it is located within the intron of the gene *EVA1C*. Two significant SNPs for NIC on chromosome 2 were annotated as intergenic variants. The SNP at position 26: 22,526,369 was significantly associated with CR42 and it is located within the intron of the candidate gene *ARMH3*.

## 4. Discussion

The present study investigated the genome-wide associations and candidate genes for milk composition and fertility traits using 42,667 SNPs from 1,537 spring-calved dairy cows in two New Zealand herds. Cows milked in both Massey dairy herds had superior fertility performance compared with the national averages for New Zealand dairy cows in the calving season from 2014–2015 and 2021–2022 [1] (SR21 = 78.1–81.3%; PRFS = 52.4–54.2%; PR42 = 65.8–67.8%). Our results for SMFS, SR21, PR21, and PR42 are in agreement with the findings by Rodriguez-Cutzal et al. [30], who reported the fertility traits of SMFS (6.5–11.3 d), SMCO (15.2–21.6 d), SR21(92–98%), PR21(48–68%), and PR42 (74–89%) for the production seasons of 2016 and 2017 in the Massey University Dairy 1 and Dairy 4 herds. We identified 40 SNPs and 73 SNPs that were significantly associated with both milk composition and fertility traits with 32 and 58 potential candidate genes at genome-wide and suggestive significant levels, respectively, in this population.

### 4.1. GWAS for Milk Composition Traits

A very significant SNP associated with the diacylglycerol O-acyltransferase 1 (*DGAT1*) gene for both FP and PP in this study is widely reported with milk yield and composition traits in several dairy cattle populations [31,32,33,34]. The *DGAT1* gene was reported to be involved in the biological functions of the monoacylglycerol biosynthetic process (GO:0006640), the triglyceride biosynthetic process (GO:0019432), lipid storage (GO:0019915), very-low-density lipoprotein particle assembly (GO:0034379), the long-chain fatty-acyl-CoA metabolic process (GO:0035336), the retinol metabolic process (GO:0042572), the diacylglycerol metabolic process (GO:0046339), the glycerolipid metabolic process (GO:0046486), and fatty acid homeostasis (GO:0055089). The *DGAT1* gene plays an important role in triacylglycerol synthesis by catalyzing the formation of an ester linkage between a fatty acyl-CoA and the free hydroxyl group of diacylglycerol [35]; triacylglycerols are the major constituent of milk fat [36].

In addition, we identified three SNPs that are close to *DGAT1* and significantly associated with FP and PP in this population. The associated candidate genes are *CPSF1, SLC52A2,* and *MROH1*. The genes *CPSF1* and *SLC52A2* are involved in mRNA polyadenylation (GO:0006378) and riboflavin transport (GO:0032218), respectively. Riboflavin is essential for the metabolism of fats and proteins. The strong association found between a riboflavin transporter gene and FP and PP suggests that the genetic control of riboflavin content is likely related to plasma transport rather than to factors related to microbial metabolism in the rumen. The biological function of the *MROH1* gene has not been discovered; the association with FP and PP was identified in previous studies [37,38]. These four candidate genes (*DGAT1, CPSF1, SLC52A2*, and *MROH1*) have been previously reported as peak variants for Fourier-transformed mid-infrared wavenumbers with highly significant protein sequence association effects for FP and PP in New Zealand dairy cattle [39]. 

Other candidate genes identified for FP and PP in this population *were IQANK1, ZC3H3,* and *RHPN1*. The gene *IQANK1* is related to the regulation of barbed-end actin filament capping (GO:2000812); this gene is a neighbour gene of *DGAT1* and has been shown to have causal effects on milk production traits independent of linkage disequilibrium [40]. The gene *ZC3H3* is involved in mRNA 3’-end processing (GO:0031124), positive regulation of the activin receptor signaling pathway (GO:0032927), and the regulation of mRNA polyadenylation (GO:1900363), whilst *RHPN1* codes negative regulation of stress fiber assembly (GO:0051497). Similar to our findings, Oliveira et al. [41] reported that the genes *ZC3H3* and *RHPN1* were significantly associated with milk yield in Canadian Holstein and Jersey cattle. We found seven genes (*DGAT1, CPSF1, SLC52A2, MROH1, IQANK1, ZC3H3,* and *RHPN1*) that are significantly associated with both FP and PP in this population. This suggests that genes that influence multiple traits are likely to be the main source of genetic correlations between traits. The results of the SNP-marker-trait associations in the present study corroborate the strong positive genetic correlation (+0.72) between FP and PP in New Zealand dairy cows [42]. 

We found four significant SNPs on chromosome 14 for FP that are located upstream of the genes *FOXH1, ADCK5, LYPD2,* and *AGO2*. The associations of these genes with FP have not been identified in the New Zealand dairy cow population previously. The *FOXH1* gene is a member of the family Forkhead box (Fox) O1, which is a primary transcription factor in glucose metabolism, in the regulation of hepatic lipid metabolism [43]. The association of *FOXH1* with milk fatty acids composition, fat yield, and FP has been well documented in previous studies [15,44,45,46]. The biological functions of the genes *ADCK5* and *LYPD2* are unknown, but Ibeagha-Awemu et al. [47] reported that the candidate gene *ADCK5* was associated with FP in Canadian Holstein cows whereas the *LYPD2* gene was associated fat yield in polish Holstein Friesian bulls [48]. The biological functions of *AGO2* are mostly related to the microRNAs (miRNAs) process (GO:0035196) and the miRNA metabolic process (GO:0010586), which regulate gene expression. A genome-wide association study by Freitas et al. [49] has shown that *AGO2* is associated with short-chain, medium-chain, long-chain, saturated, and unsaturated milk fatty acid groups in North American Holstein cattle. The candidate genes *CPSF1, SLC52A2, MROH, IQANK1, ZC3H3, RHPN1, FOXH1, ADCK5, LYPD2,* and *AGO2* found on chromosome 14 in this study could be used as a basis of linkage disequilibrium studies in the future to test whether any of these genes that neighbour *DGAT1* are associated with variation in the milk fat percentage of New Zealand dairy cattle and to test the candidate status of *DGAT1*.

The *MGST1* gene on chromosome 5 is associated with FP and has biological functions related to glutathione transport (GO:0034635), cellular oxidant detoxification (GO:0098869), and the cellular response to lipid hydroperoxide (GO:0071449), which is the highly reactive primary oxygenated product of polyunsaturated fatty acids. The functional relationship of this gene with FP has not previously been identified in New Zealand dairy cattle; however, an association study by Lopdell et al. [50] reported that *MGST1* is a strong candidate gene for lactose yield in New Zealand dairy cows. The *CSN3* associated with PP is in the casein gene family and improves milk protein quality and cheese production. This gene has biological functions related to regulating milk secretion from the mammary glands (GO:0007595) and protein stabilization (GO:0050821). We identified three SNPs on chromosomes 19 and 29 that were associated with LP. This supports previous association studies in New Zealand [39,50]. The most significant SNP was in the intron of the *PICALM* gene on chromosome 29 for LP and biological functions mostly code endocytosis activity (GO:0006897), receptor-mediated endocytosis (GO:0006898), and the regulation of endocytosis (GO:0030100). The functional relationship of the *PICALM* gene with milk protein content and cheesemaking properties has previously been reported in French dairy cows [51,52].

### 4.2. GWAS for Fertility Traits

We focused on both interval and binary fertility traits for association analysis in the present study since minimising the intervals from the start of mating to the first service and the start of mating to conception are key drivers of reproductive outcomes in seasonal calving systems. We found that the candidate gene *KCNH5* is associated with SMFS and CFS in this population, which has not been previously reported with these two traits. *KCNH5* codes ion transport (GO:0006811), potassium ion transport (GO:0006813), and potassium ion transmembrane transport (GO:0071805). Potassium is an essential micromineral component for the reproduction of early lactation dairy cows to counter metabolic acid load during heat stress [53]. 

Our results indicate that the candidate gene *HS6ST3* is associated with SMCO and FSCO. The biological function of *HS6ST3* is related to the heparan sulfate proteoglycan biosynthetic process (GO:0015012) and the heparan sulfate proteoglycan biosynthetic process with enzymatic modification (GO:0015012). Previous studies on heparan sulfate biosynthesis have revealed that heparan sulfate proteoglycans and their binding proteins play a critical role in embryonic development and growth factors [54,55,56]. Itoh and Sokol [57] demonstrated that heparan sulfate proteoglycans participate in gastrulation and mesoderm formation in the early embryo. Thus, associations of this gene with the early-stage conception-related traits of cows support the findings of improved fertility performance in this population. No previous study has identified the association of this gene with the fertility traits of dairy cows, but associations have been reported with this gene and fat yield of Italian and Swiss Brown Swiss dairy cows [58], fatty acids composition [59,60], and mastitis resistance traits in Holstein dairy cows [61].

Our results identified that four genes (*GLS, ENSBTAG00000051479, STAT1,* and *STAT4*) located on chromosome 2 are associated with SR42. However, none of those genes were associated with SR21. We do not have an explanation for this finding. The biological functions of *GLS* and *ENSBTAG00000051479* are similar and related to glutamate biosynthetic processes (GO:0006537), glutamine metabolic processes (GO:0006541), and glutamine catabolic processes (GO:0006543). Glutamine is synthesised from glutamate, via glutamine synthetase [62]. Glutamine occupies a central role linking energy and protein metabolism whereas glutamine and glutamic acid are the most abundant amino acids in milk protein [63]. The infusion of glutamine has also been shown to modulate the immune response [64]. Metabolic stress in early lactation has been identified as a risk factor associated with a delayed return to ovarian cyclicity and the decreased fertility of dairy cows [65,66]. While a direct mechanism linking glutamine with reproductive outcomes has not been identified, we know that glutamine plays a critical role as a signaling molecule in amino acid- and glucose-stimulated insulin secretion and enhancing sensitivity to insulin [67,68]. The early lactation period of dairy cattle is characterised by a severe negative energy balance, with lower blood glucose and insulin concentrations and higher blood GH concentrations [69]. We hypothesis that *GLS* and *ENSBTAG00000051479* promote increased insulin concentrations and potentially reduce the negative energy balance of dairy cows during early lactation. 

Previous literature links *STAT* genes with reproductive outcomes in dairy cattle [70,71,72]. *STAT1* and *STAT4* are two genes of the *STAT* family that code for signal transduction (GO:0007165), the regulation of transcription activity (GO:0006355), and cytokine-mediated signaling pathways (GO:0019221). *STAT* genes are the main drivers of the growth factors, cytokines, and hormones involved in reproductive processes [73]. *STAT1* plays a key role in activating the JAK/STAT signaling pathway, which regulates early embryonic development of dairy cattle [72]. *STAT* genes are involved in the regulation of implantation, establishing uterine receptivity, and regulation of the maternal immune response during pregnancy [74]. Khatib et al. [70] revealed that *STAT1* was related to fertilization and early embryonic survival rates in Holstein cattle. Associations of *STAT1* and *STAT4* with milk production traits have also been reported in Holstein dairy cows [75,76]. Further investigations with a larger dataset are warranted to determine whether any of these genes are associated with pregnancy-related traits (PR21, PR42, and PRFS) in dairy cows in seasonal calving systems.

Two candidate genes, *GPD2* and *SH3PXD2A* located on chromosomes 2 and 26, respectively, were associated with PR42 in this population. The gene *GPD2*, glycerol-3-phosphate dehydrogenase 2, catalyzes the conversion of glycerol-3-phosphate to dihydroxyacetone phosphate, which is then esterified with fatty acids to form triglycerides [77]. This gene is associated with fatty acid and triglyceride synthesis in early lactation dairy cows [77,78]. The gene *SH3PXD2A* codes in utero embryonic development (GO:0001701), the process whose specific outcome is the progression of the embryo through the uterus over time, from the formation of the zygote in the oviduct to the birth of calves. Associations of *GPD2* and *SH3PXD2A* with PR42 have not been identified in previous GWAS for fertility traits; however, Palombo et al. [79] reported that *SH3PXD2A* was associated with milk fatty acid composition in Italian Holstein dairy cows.

A single gene, *EVA1C*, was associated with PRFS in this study. The biological function of this gene is as of yet unknown. We identified one candidate gene, *ARMH3*, for the trait CR42. The biological function of *ARMH3* is related to the regulation of Golgi organization (GO:1903358), and the functions of the Golgi apparatus are the transport, sorting, and modification of both proteins and lipids [80].

The most significant SNPs for fertility traits in the current study were located on chromosome 2, in agreement with previously reported GWAS for fertility traits [16,81,82,83]. However, the candidate genes found to be associated with fertility traits show little agreement with the previously reported GWAS [84,85]. Fertility traits are polygenic in nature and also influenced by non-genetic factors, for example, the heat detection ability of the farmer. The studies reported by Minozzi et al. [84] and Parker Gaddis et al. [85] involved indoor year-around calving in Italy and US dairy cattle, whereas this study was carried out with spring-calving cows under grazing conditions.

No SNPs were significantly associated with CI, SR21, PR21, and CR21. A larger study population would increase the power to detect the significant effects associated with fertility. Although many associations were detected with milk compositional traits, associations with fertility traits are limited in this population. Fertility traits have low heritability (<10%) and the low contribution of individual QTL to the total phenotypic variance could also be attributed to the low detection of associations for fertility traits in this study. Furthermore, many significant associations detected with fertility traits were associated at the suggestive significance threshold in this study, which, on average, includes one false positive result across the genome. Lander and Kruglyak [86] documented that suggestive linkage facilitated in reporting the promising but unproven findings that were worth reporting for complex traits. In the present GWAS, the cows were genotyped using a medium density SNP panel (50K Illumina) and the study population consisted of J, F, and their crosses; however, the studied population consisted of multi-breed animals, which is a limitation of this study. The use of a high-density SNP panel would allow for less reliance on linkage disequilibrium span and structure.

## 5. Conclusions

The genome-wide association analysis in the present study detected several regions and candidate genes associated with FP, PP, LP, SMFS, SMCO, FSCO, CFS, SR42, PR42, PRFS, NIC, and CR42. The genomic regions and genes associated with milk composition traits FP, PP, and LP in this research have been identified in previous studies. We identified several genes, (*KCNH5, HS6ST3, GLS, ENSBTAG00000051479, STAT1, STAT4, GPD2,* and *SH3PXD2A*) that were significantly associated with fertility outcomes in this population. The findings of this study provide an important foundation for future genome-wide association and fine-mapping studies for fertility traits in New Zealand dairy cattle. These findings should be validated in a larger population size before they could be applied to the genomic selection of fertility traits in New Zealand dairy cattle.

## Figures and Tables

**Table 1:** Description of phenotypic (milk composition and fertility) traits used in this study.

Trait	Criteria for Trait Calculation
Milk composition	
FP	Fat percentage, kg fat per kg milk (%)
PP	Protein percentage, kg protein per kg milk (%)
LP	Lactose percentage, kg lactose per kg milk (%)
Fertility	
SMFS ^1^	The interval from the start of mating to first service (d)
SMCO ^2^	The interval from the start of mating to conception (d)
FSCO ^2^	The interval from first service to conception (d)
CFS ^1^	Interval from calving to first service (d)
CI ^3^	Interval between two consecutive calvings (d)
SR21	Cows with the first mating date in the first 21 d from the start of mating date were represented as 1, otherwise coded as 0 (binary)
SR42	Cows with the first mating date in the first 42 d from the start of mating date were represented as 1, otherwise coded as 0 (binary)
PR21	Cows conceived in the first 21 d from the start of mating date represented as 1, otherwise coded as 0 (binary)
PR42	Cows conceived in the first 42 d from the start of mating date represented as 1, otherwise coded as 0 (binary)
PRFS	Cows conceived to the first AB insemination in the first 21 d from the start of mating date were represented as 1, otherwise coded as 0 (binary)
NIC	Cows not in calf at the end of the mating period were represented as 1 and in-calf cows were coded as 0 (binary)
CR21 ^4^	Cows calved in the first 21 d from the planned start of calving date were represented as 1, otherwise coded as 0 (binary)
CR42 ^4^	Cows calved in the first 42 d from the planned start of calving date were represented as 1, otherwise coded as 0 (binary)

^1^ Intervals for cows with no recorded artificial breeding (AB) were ended at the end of the AB period of herd in each calving season (*n* = 29). ^2^ Intervals for cows that did not conceive were ended at the herd’s end of the mating date plus 21 days (*n* = 787). ^3^ Intervals for cows with positive pregnancy diagnosis results and without subsequent calving dates were calculated by estimating a calving date using the conception date plus 282 days (*n* = 1113). ^4^ Cows that did not calve in the subsequent season were treated as missing variables and both traits were not calculated for the 2021–2022 calving season.

**Table 2:** Descriptive statistics of milk composition and fertility traits of cows from Massey Dairy 1 and Dairy 4.

Trait	No. Records	Mean	SD	CV	Min.	Max.
Yields						
Milk	6381	4969	1237	24.9	387	8861
Fat	6381	232	51	21.9	22	398
Protein	6381	188	42	22.2	19	311
Lactose	6381	251	63	25.1	11	449
Milk composition						
FP%	6381	4.75	0.69	14.5	2.92	7.38
PP%	6381	3.83	0.32	8.3	2.96	5.14
LP%	6381	5.04	0.18	3.6	2.84	5.48
Fertility						
SMFS	6221	10.9	8.6	79.1	1	71
SMCO	6221	29.6	28.4	95.8	1	110
FSCO	6192	19.8	27.7	139.7	1	109
CFS	6221	79.6	18.0	22.6	14.0	201
CI	5434	368.7	21.9	5.9	261	435
SR21(%)	6221	91.4	28.0	30.7	0	1
SR42(%)	6221	99.2	8.8	8.8	0	1
PR21(%)	6221	54.1	49.8	92.0	0	1
PR42(%)	6221	75.9	42.8	56.4	0	1
PRFS(%)	6219	47.8	49.9	104.4	0	1
NIC(%)	6381	12.2	32.7	268.4	0	1
CR21(%)	4321	68.0	46.6	68.6	0	1
CR42(%)	4321	91.0	28.6	31.4	0	1

FP = fat percentage; PP = protein percentage; LP = lactose percentage; SMFS = start of mating to first service (d); SMCO = start of mating to conception (d); FSCO = first service to conception (d); CFS = calving to first service (d); CI = consecutive calving interval (d); SR21 = cow inseminated in the first 3 weeks from the start of mating; SR42 = cow inseminated in the first 6 weeks from the start of mating; PR21 = cow conceived in the first 3 weeks from the start of mating; PR42 = cow conceived in the first 6 weeks from the start of mating; PRFS = cow conceived to first service in the first 3 weeks of the breeding season; NIC = cow not in calf at the end of the breeding season; CR21 = cow calved in the first 3 weeks from the planned start of calving; CR42 = cow calved in the first 6 weeks from the planned start of calving.

**Table 3:** The genome-wide significant single nucleotide polymorphisms (SNPs) for the milk composition traits of fat percentage (FP), protein percentage (PP), and lactose percentage (LP).

Trait	Locus	Chr	Position	−log10 (*p*)	Effect (SE)	Ref./ MA	Ref. Freq.	Annotation	Gene Name
FP	rs211210569	5	93,945,738	6.76	−	C/T	0.62	Intron	*MGST1*
	rs210744919	5	93,949,810	6.43	−0.11 (0.02)	G/A	0.41	Intron	*MGST1*
	rs110984572	14	1,653,693	7.85	−0.17 (0.03)	C/T	0.89	upstream	*FOXH1*
	rs134432442	14	1,736,599	74.54	−0.33 (0.02)	C/T	0.52	missense	*CPSF1*
	rs211309638	14	1,757,935	10.01	−0.20 (0.03)	C/T	0.89	upstream	*ADCK5*
	rs137071126	14	1,765,835	84.41	−0.36 (0.02)	C/G	0.48	synonymous	*SLC52A2*
	rs109421300	14	1,801,116	89.79	−0.38 (0.02)	T/C	0.46	Intron	*DGAT1*
	rs137787931	14	1,880,378	67.95	0.32 (0.02)	T/C	0.55	Intron	*MROH1*
	rs109742607	14	2,217,163	16.15	0.16 (0.02)	A/G	0.69	Intron	*IQANK1*
	rs110323635	14	2,239,085	16.15	−0.16 (0.02)	A/G	0.31	missense	*MAPK15*
	rs109617015	14	2,386,688	17.86	−0.22 (0.03)	A/G	0.16	Intron	*ZC3H3*
	rs109529219	14	2,468,020	21.47	−0.22 (0.02)	G/A	0.22	Intron	*RHPN1*
	rs109958270	14	2,605,493	7.81	0.12 (0.02)	C/T	0.78	intergenic	*-*
	rs110043428	14	2,790,501	12.49	0.14 (0.02)	A/G	0.53	intergenic	*-*
	rs109476486	14	2,826,632	12.41	−0.17 (0.02)	T/G	0.20	upstream	*LYPD2*
	rs110545978	14	3,186,141	15.28	0.18 (0.02)	T/C	0.83	intergenic	*-*
	rs136880486	14	4,078,923	11.13	−0.13 (0.02)	T/C	0.28	upstream	*AGO2*
	rs110755656	14	5,274,635	7.65	0.11 (0.02)	G/T	0.76	intergenic	*-*
	rs110359329	14	7,428,315	6.09	−0.09 (0.02)	A/G	0.58	intergenic	*-*
PP	rs43703015	6	87,390,576	6.52	0.05 (0.01)	T/C	0.61	missense	*CSN3*
	rs43703016	6	8,7390,612	6.52	0.05 (0.01)	C/A	0.61	missense	*CSN3*
	rs110014544	6	87,390,673	6.52	0.05 (0.01)	G/A	0.61	synonymous	*CSN3*
	rs109787476	6	87,390,681	6.43	0.05 (0.01)	T/A	0.61	3 prime UTR	*CSN3*
	rs134432442	14	1,736,599	28.13	−0.09 (0.01)	C/T	0.52	missense	*CPSF1*
	rs137071126	14	1,765,835	30.83	−0.10 (0.01)	C/G	0.48	synonymous	*SLC52A2*
	rs109421300	14	1,801,116	30.96	−0.10 (0.01)	T/C	0.46	Intron	*DGAT1*
	rs137787931	14	1,880,378	23.91	0.09 (0.01)	T/C	0.55	Intron	*MROH1*
	rs109742607	14	2,217,163	6.59	0.04 (0.01)	A/G	0.69	Intron	*IQANK1*
	rs109617015	14	2,386,688	8.31	−0.07 (0.01)	A/G	0.16	Intron	*ZC3H3*
	rs109529219	14	2,468,020	9.81	−0.07 (0.01)	G/A	0.22	Intron	*RHPN1*
	rs110545978	14	3,186,141	8.33	0.06 (0.01)	T/C	0.83	intergenic	*-*
	rs110755656	14	5,274,635	5.94	0.05 (0.01)	G/T	0.76	intergenic	*-*
LP	rs378183369	29	9,563,396	8.89	−0.03 (0.004)	A/G	0.74	Intron	*PICALM*

Chr = chromosome; SE = standard error; Ref. = reference allele; MA = minor allele; Ref. freq. = reference allele frequency; FP = fat percentage; PP = protein percentage.

**Table 4:** The single nucleotide polymorphisms (SNPs) that were identified as significant at genome-wide and suggestive significance thresholds for the fertility traits.

Trait	Locus	Chr	Position	−log10 (*p*)	SNP Effect (SE)	Ref./ MA	Ref. Freq.	Annotation	Gene Name
SMFS	rs132976072	9	101,238,301	5.08	0.06 (0.01)	A/G	0.48	Intergenic	*-*
	rs110111959	10	75,105,774	5.02	0.07 (0.02)	A/G	0.75	Intron	*KCNH5*
SMCO	rs41635004	12	77,611,452	4.71	1.52 × 10^−7^ (3.55 × 10^−8^)	A/G	0.63	Intron	*HS6ST3*
FSCO	rs132906739	2	48,960,169	4.81	−0.05 (0.01)	A/G	0.57	Intergenic	*-*
	rs109941542	2	49,095,661	4.81	−0.05 (0.01)	C/T	0.57	Intergenic	*-*
	rs41635004	12	77,611,452	4.85	0.04 (0.01)	A/G	0.63	Intron	*HS6ST3*
CFS	rs132976072	9	101,238,301	5.06	0.06 (0.01)	A/G	0.48	Intergenic	*-*
	rs110111959	10	75,105,774	5.02	0.07 (0.02)	A/G	0.75	Intron	*KCNH5*
SR42	rs135632251	2	79,410,669	5.71	0.001 (0.0002)	C/T	0.94	Intergenic	*-*
	rs134983646	2	79,474,217	5.95	−0.0005 (9.82 × 10^−5^)	G/A	0.19	Intron	*GLS* *ENSBTAG00000051479*
	rs109798660	2	79,486,672	6.47	−0.001 (9.95 × 10^−5^)	T/C	0.19	Intron	*ENSBTAG00000051479*
	rs134911740	2	79,706,385	5.99	−0.0005 (9.81 × 10^−5^)	G/A	0.19	Intergenic	*-*
	rs135975975	2	79,817,588	6.52	−0.001 (9.94 × 10^−5^)	G/A	0.18	Intron	*GLS*
	rs137812009	2	79,908,334	6.50	−0.001 (9.90 × 10^−5^)	T/C	0.18	Intron	*STAT1*
	rs41610299	2	79,946,595	6.57	−0.001 (9.93 × 10^−5^)	T/C	0.18	Intron	*STAT4*
	rs380321634	2	79,975,164	5.89	0.001 (0.0002)	T/G	0.94	Intron	*STAT4*
PR42	rs137030801	2	39,681,141	5.80	4.20 × 10^−9^ (8.72 × 10^−10^)	T/C	0.46	Intron	*GPD2*
	rs109673037	26	24,432,758	4.74	0.002 (0.0004)	C/T	0.42	Upstream	*SH3PXD2A*
PRFS	rs132685083	1	2,262,097	4.93	0.004 (0.001)	A/G	0.45	Intron	*EVA1C*
NIC	rs132906739	2	48,960,169	4.86	−3.06 × 10^−8^ (7.04 × 10^−9^)	A/G	0.57	Intergenic	*-*
	rs109941542	2	49,095,661	4.86	−3.06 × 10^−8^ (7.04 × 10^−9^)	C/T	0.57	Intergenic	*-*
CR42	rs41606045	26	22,526,369	5.14	−0.003 (7.32 × 10^−6^)	C/T	0.85	Intron	*ARMH3*

Chr = chromosome; SE = standard error; Ref. = reference allele; MA= minor allele; Ref. freq. = reference allele frequency. SMFS = start of mating to first service (d); SMCO = start of mating to conception (d); FSCO = first service to conception (d); CFS = calving to first service (d); SR42 = cow inseminated in the first 6 weeks from the start of mating; PR21 = cow conceived in the first 3 weeks from the start of mating; PR42 = cow conceived in the first 6 weeks from the start of mating; PRFS = cow conceived to first service in the first 3 weeks from start of mating; NIC = cow not in calf at end of the breeding season.

## Data Availability

Data is unavailable due to ethical restrictions.

## Supplementary Materials

The following supporting information can be downloaded at: https://www.mdpi.com/article/10.3390/genes14040860/s1. Table S1: The suggestive significant single nucleotide polymorphisms (SNPs) for milk composition traits in dairy cattle.
