# Supplementary material for: Genomic Regions Associated with Milk Composition and Fertility Traits in Spring-Calved Dairy Cows in New Zealand
Source: Genes (Basel). 2023 Apr 1;14(4):860. doi: 10.3390/genes14040860 (PMC10137527; doi:10.3390/genes14040860)
Supplement: Supplementary file 1 [file genes-14-00860-s001.zip › genes-2294513-supplementary.pdf]

genes

MDPI

Table S1: The suggestive significant single nucleotide polymorphisms (SNPs) for milk composition traits in dairy cattle

Trait	Locus	Chr	Position	$-\log_{10}(P)$	Effect (SE)	Ref.	MA	Ref. freq.	Annotation	Gene name	Gene description
FP	rs134637616	5	93,945,655	5.74	0.11 (0.02)	G	T	0.66	intron	MGST1	microsomal glutathione S-transferase 1
	rs211210569	5	93,945,738	6.76	0.11 (0.02)	C	T	0.62	intron	MGST1	microsomal glutathione S-transferase 1
	rs210744919	5	93,949,810	6.43	-0.11 (0.02)	G	A	0.41	intron	MGST1	microsomal glutathione S-transferase 1
	rs41630614	14	1,514,056	5.09	0.09 (0.02)	T	G	0.68	upstream	ZNF7	zinc finger protein 7
	rs110984572	14	1,653,693	7.85	-0.17 (0.03)	C	T	0.89	upstream	FOXH1	forkhead box H1
	rs134432442	14	1,736,599	74.54	-0.33 (0.02)	C	T	0.52	missense	CPSF1	cleavage and polyadenylation specific factor 1
	rs211309638	14	1,757,935	10.01	-0.20 (0.03)	C	T	0.89	upstream	ADCK5	aarF domain containing kinase 5
	rs137071126	14	1,765,835	84.41	-0.36 (0.02)	C	G	0.48	synonymous	SLC52A2	solute carrier family 52 member 2
	rs109421300	14	1,801,116	89.79	-0.38 (0.02)	T	C	0.46	intron	DGAT1	diacylglycerol O-acyltransferase 1
	rs137787931	14	1,880,378	67.95	0.32 (0.02)	T	C	0.55	intron	MROH1	maestro heat like repeat family member 1
	rs109742607	14	2,217,163	16.15	0.16 (0.02)	A	G	0.69	intron	IQANK1	IQ motif and ankyrin repeat containing 1
	rs110323635	14	2,239,085	16.15	-0.16 (0.02)	A	G	0.31	missense	MAPK15	mitogen-activated protein kinase 15
	rs109617015	14	2,386,688	17.86	-0.22 (0.03)	A	G	0.16	intron	ZC3H3	zinc finger CCCH-type containing 3
	rs109529219	14	2,468,020	21.47	-0.22 (0.02)	G	A	0.22	intron	RHPN1	rhophilin Rho GTPase binding protein 1
	rs110060785	14	2,553,525	4.91	0.10 (0.02)	C	T	0.67	upstream	GPIHBP1	glycosylphosphatidylinositol anchored high density lipoprotein binding protein 1
	rs109958270	14	2,605,493	7.81	0.12 (0.02)	C	T	0.78	intergenic	-	-
	rs110626984	14	2,674,264	5.40	-0.11 (0.02)	T	C	0.30	intron	SLC45A4	solute carrier family 45 member 4
	rs110043428	14	2,790,501	12.49	0.14 (0.02)	A	G	0.53	intergenic	-	-
	rs109476486	14	2,826,632	12.41	-0.17 (0.02)	T	G	0.20	upstream	LYPD2	LY6/PLAUR domain containing 2
	rs134312854	14	2,898,515	4.95	-0.09 (0.02)	C	A	0.44	downstream	ARC	activity regulated cytoskeleton associated protein

Trait	Locus	Chr	Position	$-\log_{10}(P)$	Effect (SE)	Ref.	MA	Ref. freq.	Annotation	Gene name	Gene description
FP	rs110545978	14	3,186,141	15.28	0.18 (0.02)	T	C	0.83	intergenic	-	-
	rs109289626	14	3,433,372	5.78	-0.11 (0.02)	A	G	0.43	intron	TRAPPC9	trafficking protein particle complex subunit 9
	rs110461662	14	3,765,019	5.22	-0.09 (0.02)	G	A	0.58	intron	DENND3	DENN domain containing 3
	rs136880486	14	4,078,923	11.13	-0.13 (0.02)	T	C	0.28	upstream	AGO2	argonaute RISC catalytic component 2
	rs110017379	14	4,364,952	5.33	-0.09 (0.02)	T	C	0.56	intron	TRAPPC9	trafficking protein particle complex subunit 9
	rs109917734	14	4,398,699	5.60	-0.10 (0.02)	G	A	0.50	intron	TRAPPC9	trafficking protein particle complex subunit 9
	rs109086264	14	4,414,829	4.65	0.08 (0.02)	A	C	0.40	intron	TRAPPC9	trafficking protein particle complex subunit 9
	rs110755656	14	5,274,635	7.65	0.11 (0.02)	G	T	0.76	intergenic	-	-
	rs110359329	14	7,428,315	6.09	-0.09 (0.02)	A	G	0.58	intergenic	-	-
	rs109396809	19	42,493,794	4.64	0.41 (0.08)	C	T	0.41	downstream	U6	U6 spliceosomal RNA
PP	rs43703015	6	87,390,576	6.52	0.05 (0.01)	T	C	0.61	missense	CSN3	casein kappa
	rs43703016	6	8,7390,612	6.52	0.05 (0.01)	C	A	0.61	missense	CSN3	casein kappa
	rs110014544	6	87,390,673	6.52	0.05 (0.01)	G	A	0.61	synonymous	CSN3	casein kappa
	rs109787476	6	87,390,681	6.43	0.05 (0.01)	T	A	0.61	3 prime UTR	CSN3	casein kappa
	rs110398459	6	87,733,586	4.78	0.05 (0.01)	G	T	0.26	intron	ENAM	enamelin
	rs110398459	6	87,743,848	5.03	-0.04 (0.01)	A	G	0.38	intron	ENAM	enamelin
	rs134432442	14	1,736,599	28.13	-0.09 (0.01)	C	T	0.52	missense	CPSF1	cleavage and polyadenylation specific factor 1
	rs137071126	14	1,765,835	30.83	-0.10 (0.01)	C	G	0.48	synonymous	SLC52A2	solute carrier family 52 member 2
	rs109421300	14	1,801,116	30.96	-0.10 (0.01)	T	C	0.46	intron	DGAT1	diacylglycerol O-acyltransferase 1
	rs137787931	14	1,880,378	23.91	0.09 (0.01)	T	C	0.55	intron	MROH1	maestro heat like repeat family member 1
	rs109742607	14	2,217,163	6.59	0.04 (0.01)	A	G	0.69	intron	IQANK1	IQ motif and ankyrin repeat containing 1
	rs110323635	14	2,239,085	4.96	-0.04 (0.01)	A	G	0.31	missense	MAPK15	mitogen-activated protein kinase 15
	rs109617015	14	2,386,688	8.31	-0.07 (0.01)	A	G	0.16	intron	ZC3H3	zinc finger CCCH-type containing 3
	rs109529219	14	2,468,020	9.81	-0.07 (0.01)	G	A	0.22	intron	RHPN1	rhophilin Rho GTPase binding protein 1
	rs110545978	14	3,186,141	8.33	0.06 (0.01)	T	C	0.83	intergenic	-	-

Trait	Locus	Chr	Position	$-\log_{10}(P)$	Effect (SE)	Ref.	MA	Ref. freq.	Annotation	Gene name	Gene description
PP	rs136880486	14	4,078,923	5.58	-0.05 (0.01)	T	C	0.28	upstream	AGO2	argonaute RISC catalytic component 2
	rs110755656	14	5,274,635	5.94	0.05 (0.01)	G	T	0.76	intergenic	-	-
	rs109841945	14	6,276,199	4.74	-0.04 (0.01)	C	T	0.54	intergenic	-	-
LP	rs137513051	19	42,932,520	5.16	-0.03 (0.01)	T	C	0.89	intron	KCNH4	potassium voltage-gated channel subfamily H member 4
	rs136271679	19	42,988,287	4.95	-0.03 (0.01)	C	T	0.89	intron	STAT5B	signal transducer and activator of transcription 5B
	rs378183369	29	9,563,396	8.89	-0.03 (0.004)	A	G	0.74	intron	PICALM	phosphatidylinositol binding clathrin assembly protein

Chr = chromosome; SE = standard error; Ref. = reference allele; MA= minor allele; Ref. freq. = reference allele frequency; FP = fat percentage; PP = protein percentage.
